# High stone-free rate immediately after suctioning flexible ureteroscopy with Intelligent pressure-control in treating upper urinary tract calculi

**DOI:** 10.1186/s12894-022-01126-0

**Published:** 2022-11-10

**Authors:** Xingjian Gao, Zedong Zhang, Xinwei Li, Weiping Cai, Bin Zheng, Yijin Lu, Hualong Zhao, Junhong You, Gangfeng Zheng, Weilong Bao, Yutong Lai, Yisong Lv

**Affiliations:** Department of Urology, The General Hospital of Fujian Energy Group, No.18 Houxian Road, Fuzhou, 350003 Fujian Province People’s Republic of China

**Keywords:** Suctioning flexible ureteroscopy with Intelligent pressure-control, Upper urinary tract stone, Stone-free rate, Guy’s stone score, Clavien-Dindo classification

## Abstract

**Background:**

The retrospective observational study aimed to evaluate the safety and efficacy of suctioning flexible ureteroscopy with Intelligent pressure-control (SFUI) on treating upper urinary tract calculi in a large cohort.

**Methods:**

Between July 2020 and August 2021, 278 patients with upper urinary tract calculi who underwent SFUI in our hospital were enrolled. Outcomes were stone-free rate (SFR) in one session and one-month after SFUI treatment, and complications scored by the Clavien-Dindo classification.

**Results:**

A total of 310 kidneys underwent SFUI were included. The median surgery time was 75 min (ranged 60–110 min). One session and one-month SFRs were 80.65% and 82.26%, respectively. The one-session SFR was ≧ 87% in patients with Guy’s stone score of Grade I among stone size < 40 mm. Risk factors for unsuccessful stone-free in one session were stone history (adjusted odds ratio (aOR): 2.39, 95% confidence interval (CI): 1.21–4.73), stone size of 40–49 mm (aOR: 4.37, 95% CI: 1.16–16.45), Guy’s stone score ≧ Grade II (Grade II, aOR: 3.54, 95% CI: 1.18–10.59; Grade III, aOR: 10.95, 95% CI: 2.65–45.25). The incidence of Clavien-Dindo grade II-III complication was 3.26%. Complication is associated with Guy’s stone score III (aOR: 22.36, 95% CI: 1.81–276.36).

**Conclusion:**

SFUI shows good safety and efficiency on treating upper urinary tract calculi. Patients with stone size < 40 mm or Guy’s stone score of Grade I have a high chance to reach stone-free after SFUI treatment.

## Background

Upper urinary tract calculus is a common disease for global urologists. Both percutaneous nephrolithotomy (PCNL) and flexible ureteroscopy lithotripsy (FURL) are popular options for treating renal stones [1–4]. PCNL is recommended for kidney stone > 20 mm and FURL for < 20 mm in the guidelines issued by the American Urological Association [5]. FURL has high stone-free and low complication rates, however, it is easy to cause high renal pelvic pressure, resulting in complications including systemic inflammation and sepsis [6–8]. Recently, suctioning FURL has been revealed more suitable for patients with renal stones than the traditional one and acceptable in urological clinical practice [9, 10]. Besides, suctioning flexible ureteroscopy with intelligent pressure-control (SFUI), one kind of the suction FURL, was reported to provide high lithotripsy efficacy and low complication rate in treating upper urinary tract calculi for patients with a solitary kidney [9]. This retrospective study aimed to evaluate the safety and efficacy of SFUI for treating upper urinary tract calculi in a large cohort.

## Methods

### Patients

This retrospective study included patients with upper urinary tract calculi who received SFUI in our hospital between July 2020 and August 2021. All patients underwent a diagnosis of medical history, and routine preoperative examinations including urine tests, urine culture, hematology tests, kidney function tests, and kidney imaging. Patients with indications were included. No exclusion criteria. The study was reviewed and approved by the Institutional Review Board, and informed consent was waived.

Upper urinary tract stone complexity was scored by Guy’s scoring system [11]: grade 1, solitary stone in mid/lower pole or pelvis with simple anatomy; grade 2, solitary stone in the upper pole or multiple stones with simple anatomy, or a solitary stone with abnormal anatomy; grade 3, multiple stones with abnormal anatomy or stones in a calyceal diverticulum or partial stag horn calculus; grade IV, stag horn calculus or any stone with spina bifida or spinal.

## Surgery

**Fig. 1:**
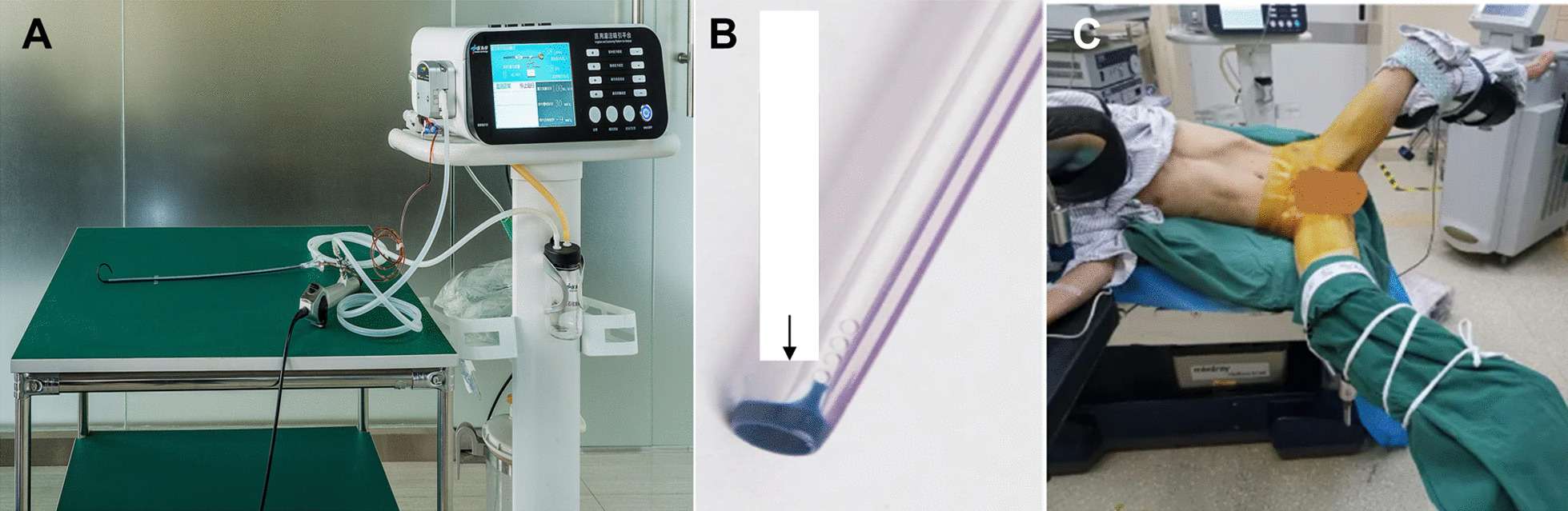
The SUFI system. **A** A patented irrigation and suction platform, consisting of a main control unit, an infusion device, a suctioning device, and a pressure feedback unit. The perfusion flow, pressure control value, and pressure limit value (30mmHg) can be monitored on the main control unit during surgery. **B** The UAS has one pressure-sensor in the front end. **C** Patient in oblique supine lithotomy position with the diseased side upward

The SFUI system contained a patented irrigation and suctioning platform and ureteral access sheath (UAS) as previously described (Fig. 1A, B) [12–14]. This system can precisely regulate the infusion flow and control the vacuum suctioning through computerized real-time recording. UAS, in which pressure sensor is located at the front end (Fig. 1B), was placed at the proximal ureter. Renal pelvic pressure was monitored by UAS and the value was shown on the system in the frequency of 6 Hz.

All surgeries were performed by one surgeon with a real-time monitor of renal pelvic pressure during the surgery. The whole flexible ureteroscopic lithotripsy procedure was performed under general anesthesia in oblique supine lithotomy position with the diseased side upward (Fig. 1C). Initial ureteroscopy was performed with a semi-rigid 8/9.8 F ureteroscope (Richard Wolf, Germany), during which a flexible 0.032-inch guidewire (Innovel, China) was inserted into the renal collecting system. Next, the patented UAS was inserted into the proximal ureter along the guidewire without fluoroscopic guidance. A disposable flexible ureteroscope (Pusen, China) was then inserted into the sheath to do a comprehensive inspection of the delivery location of the transparent sheath between mucosa of renal pelvis and ureter. After confirming that the transparent sheath is on the target position, the pressure sensory and suctioning channels were connected to the irrigation and suctioning platform. After being injected with water, zero calibration was carried out for the pressure sensory system. Fully automatic mode of the platform was then selected. Perfusion flow was set at 50-150ml/min. Renal pelvic pressure control value was set at -15–5mmHg. The maximum (limit) value was set at 30 mmHg. Intraoperatively, a holmium laser (Raykeen, China) was used to pulverize the stone at 0.8-1.6 J/pulse with a frequency of 20–30 pulses/s. In the process of lithotripsy, the scope body was moved back and forth slightly in an uninterrupted fashion in the sheath to facilitate the small gravel particles inside the sheath gap to be sucked out. Gravel particles larger than sheath gap but less than UAS in diameter were sucked out by withdrawing the scope intermittently without a need of stone basketing. For distal or mid-ureter stone, semi-rigid ureterscope was used. For patients whom the UAS was not indwelled successfully, a 7 F Double-J ureteral stent (Asymchem Inc., China) was indwelled for 2 weeks to facilitate the UAS placement for flexible ureteroscopy.

Vital signs were monitored and laboratory examinations were checked postoperatively including complete blood count, electrolytes, and calcitonin. Because repeated suction of enormous stone fragments could hurt the mucosa of ureter, a 7 F double-J ureteral stent with outer diameter F14.8 was placed at the end of the procedure and was indwelled for 4 weeks to protect the ureter, reducing the occurrence of postoperative complications such as ureter stenosis. Patients were followed at 4th week postoperatively.

## Outcome indicators

The primary outcome was stone free rate (SFR), defined as no residual stone or residual stone < 4 mm in size by X-ray image at one session and at one month after surgery, respectively. According to the Chinese Guideline for Diagnosis of Urology and Male Diseases 2019, intense follow-up is allowed for residual stone ≦ 4 mm without obstruction or infection. The secondary outcome was complications classified by Clavien-Dindo grade: grade 1, any deviation from normal postoperative course without need for pharmacological treatment, except antiemetics, antipyretics, analgetics, diuretics, electrolytes, and physiotherapy; grade 2, pharmacological treatment with drug other than such allowed for grade I, blood transfusion, and total parenteral nutrition required; grade III, surgical, endoscopic or radiological intervention required. Stone size was measured as the maximal length shown in KUB X-ray.

### Statistical analysis

Continuous data without normal distribution are presented as median (interquartile) and performed by Wilcoxon rank. Categorical data are presented as n (%) and performed by the Chi-squared test or Fisher’s exact test, as appropriate. Multiple logistic regression was adjusted for the covariates with *a p*-value < 0.1 in the univariate analysis. Data are presented as odds ratio (OR) and 95% confidence interval (CI). All *p* values are two-sided, and *p*-value < 0.05 is considered statistically significant. All statistical analyses were performed using the statistical software package SAS software version 9.4 (SAS Institute Inc., Cary, NC, USA).

## Result

A total of 278 patients were enrolled, including 310 kidneys undergoing SFUI. The baseline characteristics are presented in Table 1. The median age was 50 years (interquartile 40–59 years), with a majority of males (63.55%), median BMI of 24.8 (22.86–26.99), and 105 patients had a stone history (33.87%). The median surgery time was 75 min (60–110 min). Most patients had multiple stones (54.19%), with 75.81% stone < 30 mm, 66.13% of complex composition, 38.71% hydronephrosis Grade I and 33.87% Grade II (38.71% and 33.87%). There were 54.52% patients with Guy’s stone score of Grade I, and stone were mostly located in the ureter (46.77%) and lower segment (45.48%). The clinical outcomes are shown in Table 2. Among 310 kidneys, one session SFR and one-month SFR were 80.65% and 82.26%. Only 8 patients had Clavien-Dindo Grade II, and 2 patients had Grade III complications (ureter lesions). Twelve patients (3.87%) underwent a second stage procedure and 3 patients (0.97%) underwent third stage procedure.

**Table 1:** Characteristic

Variables	Total (n = 310)
Demography	
Age	50.00 (40.00–59.00)
Male	197 (63.55)
BMI	24.80 (22.86–26.99)
Stent in ureter	55 (17.74)
Infection	239 (77.10)
Disease history	
Stone	105 (33.87)
Other kidney diseases	19 (6.13)
DM	48 (15.48)
Hypertension	86 (27.74)
Medicine user and weakness	34 (10.97)
Operation	
Operation time	75.00 (60.00-110.00)
Stone number	
Single	142 (45.81)
Multiple	168 (54.19)
Stone size (mm)	
< 20	142 (45.81)
20–29	93 (30.00)
30–39	36 (11.61)
40–49	17 (5.48)
≥ 50	22 (7.10)
Composition	
Calcium oxalate	70 (22.58)
Calcium phosphate	23 (7.42)
Uric acid and magnesium ammonium phosphate	12 (3.87)
Complex	205 (66.13)
Hydronephrosis	
Grade 0	47 (15.16)
Grade I	120 (38.71)
Grade II	105 (33.87)
Grade III	35 (11.29)
Grade VI	3 (0.97)
CT scan	
≤ 1000	217 (70.00)
> 1000	93 (30.00)
Guy’s stone score	
Grade I	169 (54.52)
Grade II	114 (36.77)
Grade III	20 (6.45)
Grade VI	7 (2.26)
Location	
Ureter	145 (46.77)
Upper segment	20 (6.45)
Median segment	40 (12.90)
Lower segment	141 (45.48)
Multiple segment	24 (7.74)
Renal pelvis	66 (21.29)
Staghorn and full-staghorn	11 (3.55)
Anatomy abnormal	18 (5.81)

*BMI* body mass index; *DM* diabetes mellitus; *CT* computer tomography

Continuous data with normal distribution are presented a mean ± SD; Continuous data without normal distribution are presented as median (interquartile); Categorical data are presented as n (%)

**Table 2:** Clinical performance

Variables	Total (n = 310)
One-session SFR	250 (80.65)
One-month SFR	255 (82.26)
Clavien-Dindo grade (complication)	
Grade I	300 (96.77)
Grade II	8 (2.58)
Grade III	2 (0.65)
Grade VI	0 (0.00)
Grade V	0 (0.00)

*SFR* stone free rate

Continuous data with normal distribution are presented a mean ± SD; Continuous data without normal distribution are presented as median (interquartile); Categorical data are presented as n (%)

Multiple logistic regression analysis of one-session SFR is shown in Table 3. Patients with stone history (Yes: 69.52% vs. no: 86.34%, *p* < 0.001 (multiple: 69.64% vs. single: 93.66%, *p* < 0.001), stone size ≥ 40 mm (≥ 40 mm: 41.18–45.45% vs. < 40 mm: 72.22–91.55%, *p* < 0.001), and Guy’s stone score ≥ 3 (≥ 3: 40.0–42.86% vs. < 3: 71.05–93.49%, *p* < 0.001) had significantly lower chance to reach stone free in one session. Patients with one-session stone-free had less surgery time (75 vs. 99 min, *p* < 0.001). After adjusting the related variables, stone history (adjusted OR (aOR): 2.39, 95% CI: 1.21–4.73), large stone size (40–49 mm: aOR = 4.37, 95% CI = 1.16–16.45, compared to stone < 20 mm), and high Guy’s stone score (Grade III: aOR = 10.95, 95% CI: 2.65–45.25; Grade II: aOR = 3.54, 95% CI: 1.18–10.59, compared to Grade I respectively) still displayed significantly higher risks of not completely cleaned stone in one session.

**Table 3:** The comparison of one session stone free rate (SFR) and estimated OR for non-one session stone free

Variables	One session stone free		*p*-value	Multivariate analysis
	Yes (n = 250)	No (n = 60)		aOR (95%CI)
*Demography*				
Age	50 (42.00–59.00)	46.5 (35.50–58.00)	0.176	–
Gender			0.525	–
Male	161 (81.73)	36 (18.27)		
Female	89 (78.76)	24 (21.24)		
BMI	24.81 (22.86–26.99)	24.42 (22.90–26.93)	0.634	–
Stent in ureter			0.320	–
Yes	47 (85.45)	8 (14.55)		
No	203 (79.61)	52 (20.39)		
Infection			0.105	–
Yes	188 (78.66)	51 (21.34)		
No	62 (87.32)	9 (12.68)		
*Disease history*				
Stone			**< 0.001**	**2.39 (1.21, 4.73)**
Yes	73 (69.52)	32 (30.48)		
No	177 (86.34)	28 (13.66)		
Other kidney diseases			0.770^a^	–
Yes	15 (78.95)	4 (21.05)		
No	235 (80.76)	56 (19.24)		
DM			0.363	–
Yes	41 (85.42)	7 (14.58)		
No	209 (79.77)	53 (20.23)		
Hypertension			0.597	–
Yes	71 (82.56)	15 (17.44)		
No	179 (79.91)	45 (20.09)		
Medicine user and weakness			0.847	-
Yes	27 (79.41)	7 (20.59)		
No	223 (80.8)	53 (19.2)		
*Operation*				
Operation time	75 (60.0–105.0)	99 (70.0–144.0)	**< 0.001**	1.01 (1.00, 1.01)
Stone number			**< 0.001**	
Single	133 (93.66)	9 (6.34)		1
Multiple	117 (69.64)	51 (30.36)		1.11 (0.34, 3.60)
Stone size(mm)			**< 0.001**	
< 20	130 (91.55)	12 (8.45)		1
20–29	77 (82.80)	16 (17.20)		1.34 (0.54, 3.36)
30–39	26 (72.22)	10 (27.78)		1.67 (0.53, 5.26)
40–49	7 (41.18)	10 (58.82)		**4.37 (1.16, 16.45)**
≥ 50	10 (45.45)	12 (54.55)		2.41 (0.58, 9.96)
Composition			0.368	
Calcium oxalate	55 (78.57)	15 (21.43)		–
Calcium phosphate	18 (78.26)	5 (21.74)		–
Uric acid and magnesium ammonium phosphate	12 (100.00)	0 (0.00)		–
Complex	165 (80.49)	40 (19.51)		–
Hydronephrosis			0.806	
Grade 0	38 (80.85)	9 (19.15)		–
Grade I	97 (80.83)	23 (19.17)		–
Grade II	87 (82.86)	18 (17.14)		–
Grade III	26 (74.29)	9 (25.71)		–
Grade VI	2 (66.67)	1 (33.33)		–
CT value (ml)			0.06	
≤ 1000	181 (83.41)	36 (16.59)		1
> 1000	69 (74.19)	24 (25.81)		1.22 (0.59, 2.52)
Guy’s stone score			**<0.001**^**a**^	
Grade I	158 (93.49)	11 (6.51)		1
Grade II	81 (71.05)	33 (28.95)		**3.54 (1.18, 10.59)**
Grade III	8 (40.00)	12 (60.00)		**10.95 (2.65, 45.25)**
Grade VI	3 (42.86)	4 (57.14)		2.18 (0.23, 21.21)

The significance of bold values were* p* < 0.05

*SFR* stone free rate, *aOR* adjusted Odds ratio, *BMI* body mass index, *DM* diabetes mellitus; *CT* computer tomography

Continuous data without normal distribution are presented as median (interquartile) and performed by Wilcoxon rank; Categorical data are presented as n (%) and performed by the Chi-squared test or ^a^Fisher’s exact test, as appropriate

Multiple logistic regression analysis of one-month SFR is shown in Table 4. The results of one-month SFR were similar with one-session SFR. After adjusting the related variables, stone history (aOR: 2.48, 95% CI: 1.24–4.97) and Guy’s stone score (Grade II, aOR: 4.44, 95% CI: 1.35–14.58; Grade III, aOR: 15.44, 95% CI: 3.48–68.55, compared to Grade I respectively) displayed significantly higher risks of not completely cleaned stone in one-month after SUFI. Besides, although there was no significant association between hydronephrosis and one session or one-month SFR, SFR was reduced as the hydronephrosis grade elevated.

**Table 4:** The comparison of one-month stone free rate (SFR) and estimated OR for non-one-month SFR

Variables	One-month stone free		*p*-value	Multivariate analysis
	Yes (n = 255)	No (n = 55)		aOR (95%CI)
*Demography*				
Age	50 (42.00–60.00)	45 (35.00–57.00)	0.086	0.99 (0.96, 1.01)
Gender				
Male	162 (82.23)	35 (17.77)	0.988	–
Female	93 (82.3)	20 (17.7)		
BMI	24.82 (22.86–27.06)	24.22 (22.30–26.81)	0.407	–
Stent in ureter			0.494	–
Yes	47 (85.45)	8 (14.55)		
No	208 (81.57)	47 (18.43)		
Infection			0.203	–
Yes	193 (80.75)	46 (19.25)		
No	62 (87.32)	9 (12.68)		
*Disease history*				
Stone			**< 0.001**	**2.48 (1.24, 4.97)**
Yes	75 (71.43)	30 (28.57)		
No	180 (87.8)	25 (12.2)		
Other kidney diseases			0.756^a^	–
Yes	15 (78.95)	4 (21.05)		
No	240 (82.47)	51 (17.53)		
DM			0.533	–
Yes	41 (85.42)	7 (14.58)		
No	214 (81.68)	48 (18.32)		
Hypertension			0.157	–
Yes	75 (87.21)	11 (12.79)		
No	180 (80.36)	44 (19.64)		
Medicine user and weakness			0.645	–
Yes	27 (79.41)	7 (20.59)		
No	228 (82.61)	48 (17.39)		
*Operation*				
Operation time	75 (60.0–105.0)	100 (65.0–150.0)	**0.002**	1.01 (1.00, 1.01)
Stone number			**< 0.001**	
Single	134 (94.37)	8 (5.63)		1
Multiple	121 (72.02)	47 (27.98)		0.99 (0.28, 3.48)
Stone size(mm)			**< 0.001**	
< 20	130 (91.55)	12 (8.45)		1
20–29	80 (86.02)	13 (13.98)		1.00 (0.38, 2.62)
30–39	26 (72.22)	10 (27.78)		1.57 (0.49, 5.07)
40–49	8 (47.06)	9 (52.94)		2.91 (0.76, 11.11)
≥ 50	11 (50.00)	11 (50.00)		1.58 (0.37, 6.84)
Composition			0.467^a^	
Calcium oxalate	58 (82.86)	12 (17.14)		–
Calcium phosphate	19 (82.61)	4 (17.39)		–
Uric acid and magnesium ammonium phosphate	12 (100.00)	0 (0.00)		–
Complex	166 (80.98)	39 (19.02)		–
Hydronephrosis			0.955	
Grade 0	39 (82.98)	8 (17.02)		–
Grade I	99 (82.50)	21 (17.50)		–
Grade II	87 (82.86)	18 (17.14)		–
Grade III	28 (80.00)	7 (20.00)		–
Grade VI	2 (66.67)	1 (33.33)		–
CT value (ml)			0.144	
≤ 1000	183 (84.33)	34 (15.67)		–
> 1000	72 (77.42)	21 (22.58)		–
Guy’s stone score			**< 0.001** ^**a**^	
Grade I	160 (94.67)	9 (5.33)		1
Grade II	84 (73.68)	30 (26.32)		**4.44 (1.35, 14.58)**
Grade III	8 (40.00)	12 (60.00)		**15.44 (3.48, 68.55)**
Grade VI	3 (42.86)	4 (57.14)		2.93 (0.28, 30.29)

The significance of bold values were* p* < 0.05

*SFR* stone free rate; *aOR* adjusted Odds ratio, *BMI* body mass index, *DM* diabetes mellitus, *CT* computer tomography

Continuous data without normal distribution are presented as median (interquartile) and performed by Wilcoxon rank; Categorical data are presented as n (%) and performed by the Chi-squared test or ^a^Fisher’s exact test, as appropriate

Multiple logistic regression analysis of complication classified by Clavien-Dindo grade is shown in Table 5. Patients with multiple stone number (5.95% vs. single: 0%, *p* = 0.002), big stone size (≥ 50 mm: 13.64% vs. other size: 1.08–5.88%, *p* = 0.028), and higher Guy’s stone score (Grade I to VI: 0.59–14.29%, *p* = 0.002) had higher proportion to reach Grade II-IV. After adjusting for the stone size, Guy’s stone score remained significantly correlated to complications (Grade III vs. Grade I: aOR = 22.36, 95% CI = 1.81-276.36).

**Table 5:** The comparison of complication (Clavien-Dindo grade) and estimated OR for Grade II-IV.

Variables	Clavien-Dindo grade		*p*-value	Multivariate analysis
	Grade I (n = 300)	Grade II-IV (n = 10)		aOR (95%CI)
*Demography*				
Age	50 (40.00–59.00)	44.5 (39.00–58.00)	0.603	–
Gender				
Male	193 (97.97)	4 (2.03)	0.179	–
Female	107 (94.69)	6 (5.31)		
BMI	24.79 (22.85–26.82)	26.22 (23.88–27.28)	0.259	–
Stent in ureter			1.000^a^	–
Yes	54 (98.18)	1 (1.82)		
No	246 (96.47)	9 (3.53)		
Infection			0.701^a^	–
Yes	232 (97.07)	7 (2.93)		
No	68 (95.77)	3 (4.23)		
*Disease history*				
Stone			0.738^a^	–
Yes	101 (96.19)	4 (3.81)		
No	199 (97.07)	6 (2.93)		
Other kidney diseases			1.000^a^	–
Yes	19 (100.00)	0 (0.00)		
No	281 (96.56)	10 (3.44)		
DM			0.190^a^	–
Yes	45 (93.75)	3 (6.25)		
No	255 (97.33)	7 (2.67)		
Hypertension			0.110^a^	–
Yes	81 (94.19)	5 (5.81)		
No	219 (97.77)	5 (2.23)		
Medicine user and weakness			0.301^a^	–
Yes	32 (94.12)	2 (5.88)		
No	268 (97.1)	8 (2.9)		
*Operation*				
Operation time	75 (60.0–109.5)	82.5 (40.0–185.0)	0.768	–
Stone number			**0.002** ^a^	
Single	142 (100.00)	0 (0.00)		–
Multiple	158 (94.05)	10 (5.95)		–
Stone size (mm)			**0.028** ^a^	
< 20	139 (97.89)	3 (2.11)		1
20–29	92 (98.92)	1 (1.08)		0.32 (0.03, 3.44)
30–39	34 (94.44)	2 (5.56)		1.02 (0.15, 7.19)
40–49	16 (94.12)	1 (5.88)		0.73 (0.06, 8.64)
≥ 50	19 (86.36)	3 (13.64)		1.75 (0.25, 12.35)
Composition			0.278^a^	
Calcium oxalate	67 (95.71)	3 (4.29)		–
Calcium phosphate	21 (91.30)	2 (8.70)		–
Uric acid and magnesium ammonium phosphate	12 (100.00)	0 (0.00)		–
Complex	200 (97.56)	5 (2.44)		–
Hydronephrosis			0.772 ^a^	
Grade 0	45 (95.74)	2 (4.26)		–
Grade I	115 (95.83)	5 (4.17)		–
Grade II	102 (97.14)	3 (2.86)		–
Grade III	35 (100.00)	0 (0.00)		–
Grade VI	3 (100.00)	0 (0.00)		–
CT value (ml)			0.494 ^a^	
≤ 1000	211 (97.24)	6 (2.76)		–
> 1000	89 (95.70)	4 (4.30)		–
Guy’s stone score			**0.002** ^**a**^	
Grade I	168 (99.41)	1 (0.59)		1
Grade II	109 (95.61)	5 (4.39)		8.11 (0.79, 82.96)
Grade III	17 (85.00)	3 (15.00)		**22.36 (1.81, 276.36)**
Grade VI	6 (85.71)	1 (14.29)		15.90 (0.55, 456.26)

The significance of bold values were* p* < 0.05

*aOR* adjusted Odds ratio, *BMI* body mass index, *DM* diabetes mellitus, *CT* computer tomography

Continuous data without normal distribution are presented as median (interquartile) and performed by Wilcoxon rank; Categorical data are presented as n (%) and performed by the Chi-squared test or ^a^Fisher’s exact test, as appropriate

Table 6 lists the one-session SFR in Guy’s stone score grade and stone size category. One-session SFR was higher than 87% in Guy’s stone score Grade I among all included stone sizes. However, one-session SFR was less than 80% when Guy’s stone score Grade ≧ II.

**Table 6:** One session stone free rate (SFR) in Guy’s stone score grade and stone size category

Stone size (mm)	Guy’s stone score			
	Grade I	Grade II	Grade III	Grade IV
	One session stone free/N (%)			
< 20	108/112 (96.43)	19/24 (79.17)	3/6 (50.00)	0/0 (–)
20–29	41/47 (87.23)	35/45 (77.78)	1/1 (100.00)	0/0 (–)
30–39	7/8 (87.50)	17/24 (70.83)	2/4 (50.00)	0/0 (–)
40–49	1/1 (100.00)	4/10 (40.00)	1/4 (25.00)	1/2 (50.00)
≥ 50	1/1 (100.00)	6/11 (54.55)	1/5 (20.00)	2/5 (40.00)

*SFR* stone free rate

## Discussion

This study demonstrated good safety and efficacy of SFUI, with one-session SFR of 80.65% (250/310) and a low complication rate (3.26%). Patients with stone size < 40 mm or Guy’s stone score of Grade I had a significantly higher potential to reach stone-free after SFUI treatment. The unique advantage of SFUI is to clear renal stone with real-time monitoring RPP and increased perfusion flow to kidney safely, solving the very gap of moving out pulverized stone under FURL treatment. It surmounts the limitation of clearing kidney stone > 20 mm through urinary tract and displays high one-session SFR with rare complications. Therefore, SFUI has a potential to be an option for patients with large kidney stones for diminished injury and faster recovery.

The present study showed the one-session SFR ≧ 87% among patients with Guy’s stone score of Grade I among all included stone sizes, and few postoperative complications of Clavien-Dindo grade ≧ Grade II. This suggests that SFUI is optimal for patients with Guy’s stone score of Grade I regardless of stone size. It was reported that the SFR with FURL treatment reached > 95% for stone ≦ 2 cm [15, 16], but significantly reduced to ~ 60% for stone > 2 cm [14–16] or required more procedures [17, 18]. SFR of FURL reduced as stone size increased. Meta-analysis studies reported that the SFR of PCNL was significantly higher than FURL, while the safety of FURL was higher than PCNL [19, 20]. However, comparative nonrandomized studies of small cohort reported comparable complication rate and comparable SFR of FURL and PCNL for stone ≦ 3 cm [21, 22], but a significantly higher SFR in PCNL than FURL for stone > 3 cm [19]. Compared to FURL and PCNL, SFUI displayed good one-session SFR with rare postoperative complications in all included stone sizes.

Our results showed significantly negative correlation of Guy’s stone score with SFRs and complications. Therefore, Guy’s stone score could be used as a predictive factor of SFR and complications for patients received SUFI. Guy’s stone score is a widely used validated scoring system to provide standardized information of stone complexity and treatment outcomes [23]. There are several scoring systems assessing the complexity of stones, including Guy’s stone score, S.T.O.N.E., and CROES. Each has advantages and disadvantages, and no one is regard as the very golden standard. Studies showed all these systems have comparable ability in predicting stone-free status [23–26]. More studies are needed to clarify clinical factors associated with outcomes in different patient populations.

Preoperative hydronephrosis was reported to affect the outcome of FURS and PCNL [27, 28]. A case-control study with 66 patients revealed that hydronephrosis might significantly affect the SFR of micropercutaneous nephrolithotomy (microperc), a new minimal-invasive technique [27]. Another retrospective study with 164 patients revealed that the success of FURS might decrease as Grade II or a more severe grade of hydronephrosis [28]. In this study, no association between SFR and hydronephrosis was found; however, one-month SFR was reduced following the elevation of hydronephrosis grade. In addition, studies suggested non-contrast CT detected stone density > 1000 HU as a significant predictor of stone fragment failure by extracorporeal shock wave lithotripsy [29, 30]. A tendency toward not completely cleaned stone in one session after SFUI for CT > 1000 HU was found in our study, implying more therapeutic protocols after SFUI may be needed to clean residual stone fragments in upper urinary tract.

### Limitation

There are some limitations in this study. First, this is a retrospective study with the inherent limitation. Secondly, the lack of a control group or another surgical method might lead to the bias of data interpretation. Third, postoperative KUB may overestimate the true SFR.

## Conclusion

Patients undergoing SFUI showed good stone-free rate and rare complications. One-session SFR is > 87% in patients with Guy’s stone score I, while high Guy’s stone score is associated with decreased one-session SFR and complications. Patients with stone size < 40 mm or Guy’s stone score of Grade I have a high chance to reach stone free after SFUI treatment.

## Data Availability

All data analysed during this study are included in this published article.

## Abbreviations

SFUI: Suctioning flexible ureteroscopy with intelligent pressure-control
SFR: Stone-Free Rate
PCNL: Percutaneous nephrolithotomy
FURL: Flexible ureteroscopy lithotripsy
aOR: Adjusted odds ratio
CI: Confidence interval
